# Emphasizing the diagnostic and management challenge: Nodular sclerosis Hodgkin lymphoma with pericardial infiltration in an adolescent male - A case report

**DOI:** 10.1016/j.lrr.2026.100596

**Published:** 2026-05-28

**Authors:** Saja I. AbuGhannam, Celina R. Andonie, Leen I. Masalmeh, Akram Karama, M.Ziad Alresheq, Aliaa’ khalili

**Affiliations:** a Al-Quds University, Jerusalem, Palestine; b Nuclear Medicine Department, Al-Ahli Hospital, Hebron, Palestine; c Hemato-oncology department, Dura Hospital, Hebron, Palestine

**Keywords:** Hodgkin, Lymphoma, Pericardial infiltration

## Abstract

Hodgkin’s lymphoma is a malignancy of lymphoid tissue with a generally favorable prognosis; however, symptomatic initial presentation with pericardial involvement is rare and presents significant diagnostic and therapeutic challenges. We report the case of a previously healthy 17-year-old male who presented with a four-month history of progressive cough and exertional dyspnea, along with a newly noticed right supraclavicular mass, without systemic B symptoms. Transthoracic echocardiography demonstrated a mild pericardial effusion, and subsequent PET-CT imaging revealed findings consistent with advanced disease. An excisional biopsy of the lymph node confirmed the diagnosis of nodular sclerosis classical Hodgkin lymphoma, and the patient was staged as Stage IV due to both pulmonary and pericardial involvement. Initial cytoreductive treatment with COP (Cyclophosphamide, Vincristine, Prednisone) was administered, followed by standard ABVD (Adriamycin, Bleomycin, Vinblastine, Dacarbazine) chemotherapy. This case illustrates the importance of including lymphoproliferative disorders in the differential diagnosis of pericardial effusion. Furthermore, it emphasizes the importance of early imaging, histopathological confirmation, and a multidisciplinary approach in guiding appropriate treatment strategies. As this can help prevent severe complications and enhance the patient's prognosis.

## 1 Introduction

Hodgkin’s lymphoma (HL) is a neoplasm of lymphoid tissue that is classified according to the WHO Classification of Hematolymphoid Tumors (5th edition, 2022) and The International Consensus Classification (ICC, 2022) into two main entities: classic Hodgkin lymphoma (cHL) and nodular lymphocyte-predominant lymphoma. According to the WHO classification, the latter is termed nodular lymphocyte-predominant Hodgkin lymphoma, whereas the ICC classification refers to it as nodular lymphocyte-predominant B-cell lymphoma (NLPBL). Classic Hodgkin lymphoma is further subdivided into nodular sclerosis, mixed cellularity, lymphocyte-rich, and lymphocyte-depleted subtypes [1,2].

Cardiac involvement in HL is infrequently reported in the literature and is possibly due to direct malignant infiltration of the pericardium [3,4] Pericardial effusion in patients with HL is typically asymptomatic and rarely represents the initial presentation, occurring in only about five percent of cases [5,6] It may result from direct malignant involvement of the pericardium or from indirect mechanisms such as obstruction, inflammation or irritation, immune-mediated processes, or other contributing factors [3,4].

HL is a malignancy with a favorable prognosis and high potential for cure. It has diverse clinical manifestations and may include constitutional symptoms such as fever, unintentional weight loss, and night sweats, or it may be identified incidentally through asymptomatic lymphadenopathy. In some cases, a mediastinal mass may cause localized or systemic effects. Although uncommon, HL can also present with symptomatic pericardial effusion, as observed in the current case. Patients with HL are typically at risk of developing secondary malignancies such as lung cancer, acute leukemia, and non-Hodgkin lymphoma, as well as cardiovascular complications beyond pericardial involvement. The presentation of symptomatic pericardial effusion as the initial manifestation of HL, as observed in this case, is particularly uncommon. While pericardial effusion may develop later in the disease course, it can also occur as an adverse effect of therapy, particularly following radiation treatment. Therefore, early detection and timely intervention are important to prevent progression to life-threatening complications such as cardiac tamponade [7].

This case report describes a 17-year-old male who presented with a four-month history of progressive cough and dyspnea, with painless swelling in the right supraclavicular region. Transthoracic echocardiography revealed the presence of a minimal pericardial effusion. Further laboratory investigations and imaging studies led to detection of infiltration of the pericardium near the root of the great vessels and eventually diagnosis of HL. This case hopes to emphasize the importance of including lymphoproliferative disorders in the differential diagnosis of pericardial effusion, particularly in young patients presenting with nonspecific cardiopulmonary symptoms. Early recognition and timely management are essential to prevent potentially life-threatening complications and to improve overall prognosis.

## 2 CASE presentation

A previously healthy 17-year-old male admitted to the hospital complaining of worsening exertional dyspnea and a persistent non-productive cough for the past four months. During this time, he also observed a painless swelling in the right supraclavicular area. He denied any systemic 'B' symptoms such as fever, night sweats, or unintended weight loss. Upon physical examination, the patient appeared clinically stable with normal vital signs. However, a painless mass in the right supraclavicular region was noted during the examination. There was no evidence of additional peripheral lymphadenopathy or hepatosplenomegaly.

Initial laboratory tests indicated slightly elevated WBC (12.9K/ microL), predominant neutrophilia (92.8%), with a significant drop in lymphocytes (6.7%). This contributed to an International Prognostic Score (IPS) of 3 (IPS included age, gender, albumin, stage III/IV, WBCs greater or equal to 15 K, and lymphocyte count <600/mm^3^ or <8% of WBCs). Both hemoglobin and hematocrit levels were normal. The coagulation profile showed an elevation in prothrombin time (PT 16.78 *sec*). Additionally, albumin was normal (4.50 g/dL), other liver and kidney function tests returned normal. The ECG was unremarkable, with no evidence of pericarditis related changes.

**Fig. 1:**
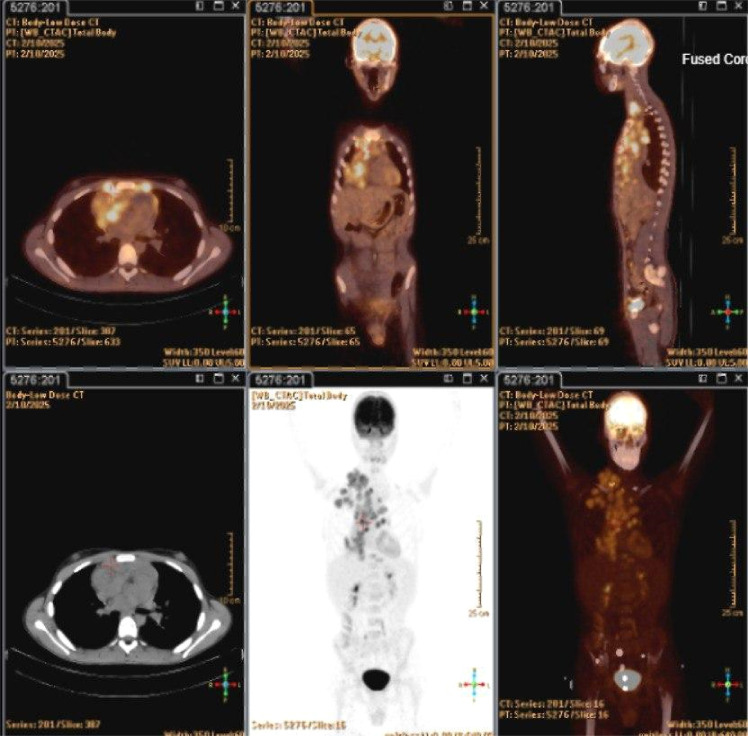
There appears to be a large anterior mediastinal mass occupying a significant portion of the mediastinum, compressing or displacing surrounding structures. The lungs (especially on the left side of the image, corresponding to the patient’s right lung) appear compressed, while the right lung field (on the left side of the image) seems relatively expanded and air-filled. The mass shows a heterogeneous appearance — meaning it’s not uniformly dense — which could suggest necrosis, hemorrhage, or mixed solid and cystic components.

Staging investigations using PET-CT revealed hypermetabolic lymphadenopathy above the diaphragm, a right pulmonary nodule with significant FDG uptake (SUV 6.7) and moderate FDG-avid infiltration of the pericardium near the root of the great vessels (SUV 3.7), see Fig. 1. Therefore, an excisional biopsy of the supraclavicular lymph node confirmed the diagnosis of nodular sclerosis classic Hodgkin's lymphoma, classified as stage IV due to the presence of extra-nodal pulmonary and pericardial involvement.

Transthoracic echocardiography was done, which showed a minimal pericardial effusion, and borderline left ventricular dilation with a preserved left ventricular ejection fraction (70%). Given the cardiac findings and the risk of anthracycline toxicity, the patient was transferred to a tertiary care center for close observation, in addition, sperm cryopreservation was done prior to the commencement of chemotherapy.

The initial cytoreductive treatment began with COP (Cyclophosphamide, Vincristine, Prednisone) with follow up of pro-BNP. After the COP therapy, the patient's Pro-BNP level rose significantly, leading to the initiation of Colchicine (0.5 mg twice daily) to address pericardial inflammation. Then, a first cycle of ABVD regimen (Adriamycin, Bleomycin, Vinblastine, and Dacarbazine) was administered without any complications, with subsequent Pro-BNP levels reduced. The patient was discharged in good overall condition with a plan to continue treatment, which included Brentuximab Vedotin combined with AVD (BV-AVD) to improve progression-free survival in patients with advanced-stage disease (stage III/IV).

## 3 Discussion

Malignant pericardial involvement occurs in approximately 21% of cancer patients. Typically, at an advanced stage of the disease and is generally associated with poor outcomes [6] The cancers most frequently spreading to the heart or pericardium include lung cancer, breast cancer, lymphoma, and melanoma. The pericardium is the most frequent cardiac site for tumor involvement [6] Approximately 25% of patients with disseminated (advanced/widespread) lymphoma may develop cardiac involvement [8] Research by Roberts et al. and Cairns et al. indicates that lymphoma accounts for around 9% of all cardiac tumors [[9], [10], [11]].

Pericardial effusion, in patients with diagnosed malignancy, may be caused by pericardial involvement secondary to the underlying malignancy, direct invasion from primary cardiac tumors, infections, idiopathic pericarditis, or drug- or radiation-induced pericarditis [5] Additionally, immunocompromised patients undergoing chemotherapy may develop autoimmune pericardial effusions [7] In lymphoma, pericardial effusion is frequently caused by lymphatic and venous drainage system involvement [6,12,13] The blockage of the visceral pericardium lymphatic channels, that drains the pericardial space, by malignant deposits or enlarged lymph nodes leads to pericardial effusion. Interestingly, effusion can also occur without direct pericardial involvement [6,7].

Pericardial effusion in HL is rare, affecting only about 5–6% of patients, and it is rarely the initial presentation of the disease [12] Most patients with cardiac involvement remain clinically silent. However, when symptoms occur, dyspnea and cough are most frequently reported, with dysphagia, hoarseness, and pleuritic chest pain also noted [6,7,14] The clinical presentation depends on how quickly pericardial fluid accumulates; rapid accumulation of even 250 ml can lead to cardiac tamponade [7].

In a retrospective study consisting 273 patients by Bashir et al., a significant association (p-value of 0.02) was identified between nodular sclerosis classical Hodgkin lymphoma and pericardial involvement [6] In this study two patients had pericardial drainage but no cytomorphology workup of this fluid was done. Both the reported study [6] and our case have some area of uncertainty; the nature of the effusion in CHL remains incompletely defined. While tumor-related or inflammatory mechanisms would typically suggest an exudative process, alternative mechanisms such as lymphatic obstruction may also play a significant role [15] Furthermore, in nodular sclerosis CHL, the presence of mediastinal fibrosis may alter local fluid dynamics and, in some cases, may present in transudate like effusion [6].

In the present case, pericardiocentesis was not performed due to the minimal volume of effusion and the absence of hemodynamic compromise. While cytomorphological analysis of pericardial fluid can provide valuable diagnostic information, particularly in distinguishing malignant from reactive processes, its role remains context-dependent and should be guided by clinical indication [16] Cytologic examination of pericardial fluid has demonstrated a high sensitivity (92.1%) for diagnosing malignancy, significantly outperforming pericardial biopsy (55.3%) [16] However, in patients without hemodynamic instability, conservative management with close observation is often appropriate [6].

This lymphoma subtype typically presents with a bulky mediastinal mass exceeding one-third of the maximum thoracic diameter [6] Contagious spread to adjacent thoracic structures, including the pleura, pericardium, or chest wall, generally occurs only after the anterior mediastinal or paratracheal mass has grown beyond 30% of the thoracic diameter [6,17].

While echocardiography is highly sensitive for detecting pericardial effusion, CT imaging is better in assessing tumor involvement of the pericardium and cardiac tissue [6,12,18] Also, CT can detect nodular thickening of the pericardium even when no effusion is present [12,18] Usually, the underlying cause of pericardial effusion can be presumed to be related to an underlying condition. However, when in doubt, pericardiocentesis should be performed to establish a definitive diagnosis [6].

This case is unique due to its rare first presentation as our patient was admitted because he was complaining of cough and dyspnea for 4 months with new onset right supraclavicular mass with no B symptoms. PET CT showed LAP above the diaphragm, and hypermetabolic right pulmonary nodule and hypermetabolic pericardium infiltration, cardiac echo showed mild pericardial effusion with LV is borderline dilated and excisional biopsy from the right supraclavicular mass: classical HL, nodular sclerosing subtype. So, our patient was diagnosed with stage 4 classical nodular sclerosing HL, with worse prognosis according to the International Prognostic Score (IPS) (IPS of 3 points; male, stage 4 disease and lymphocyte% <8% of the total WBC count).

Treatment of HL with pericardial involvement requires a multidisciplinary approach involving hematology-oncology, cardiology, cardio-thoracic surgery, radiology and radiation-oncology, and importantly laboratory medicine including hematopathology and immunophenotypic analysis. For patients with life-threatening pericardial effusions, urgent pericardiocentesis is recommended, followed by chemotherapy. A pericardial window may be considered an option for long-term drainage due to the high-risk of recurrence [19] Standard treatment of HL consists of six cycles of combination chemotherapy, with interim PET scanning after two cycles (PET-2) to guide further management [20] ABVD remains a widely accepted first-line regimen, especially for earlier stages (stage 1 and 2), due to its lower acute and long-term toxicity profile, especially when compared to the escalated BEACOPP regimen (Bleomycin, Etoposide, Adriamycin, Cyclophosphamide, Vincristin, Procarbazine, Prednisolone) [19,20] For advanced HL (stages 3 and 4) brentuximab-AVD regimen is used as frontline option for treatment, especially in patients at risk for bleomycin-induced toxicity. A recent randomized control study (SWOG S1826) found that nivolumab-AVD is superior to brentuximab-AVD [19].

Even though pericardial effusion often presents without symptoms and improves during chemotherapy, it is considered as an independent indicator for poor outcomes in children and adolescents with intermediate-risk HL. According to Cox regression survival analysis, the presence of pericardial effusion is a significant factor affecting event-free survival, even with considering the other risk factors [21].

## 4 Conclusion

In conclusion, this case report emphasizes the need for heightened awareness regarding the atypical presentation of HL, particularly in the context of pericardial effusion. This case contributes to the existing literature as a reminder for physicians and clinicians to consider lymphoproliferative disorders in the differential diagnosis when encountering unexplained pericardial effusion. Keeping in mind that addressing HL proactively and early can enhance the patient’s prognosis, and minimize the risk of severe complications associated with delayed diagnosis.

## CRediT authorship contribution statement

**Saja I. AbuGhannam:** Writing – review & editing, Writing – original draft, Visualization, Validation, Data curation, Conceptualization. **Celina R. Andonie:** Writing – review & editing, Writing – original draft, Visualization, Validation, Formal analysis, Data curation, Conceptualization. **Leen I. Masalmeh:** Writing – review & editing, Writing – original draft, Visualization, Validation, Formal analysis, Data curation, Conceptualization. **Akram Karama:** Visualization, Validation, Supervision, Investigation, Conceptualization. **M.Ziad Alresheq:** Writing – original draft. **Aliaa’ khalili:** Writing – review & editing, Visualization, Validation, Supervision, Investigation, Conceptualization.

## Declaration of competing interest

The authors declare that they have no known competing financial interests or personal relationships that could have appeared to influence the work reported in this paper.
